# Efficient broadband light absorption in thin-film a-Si solar cell based on double sided hybrid bi-metallic nanogratings

**DOI:** 10.1039/c9ra10232a

**Published:** 2020-03-24

**Authors:** Fazal E. Subhan, Aimal Daud Khan, Fazal E. Hilal, Adnan Daud Khan, Sultan Daud Khan, Rehan Ullah, Muhammad Imran, Muhammad Noman

**Affiliations:** Center for Advanced Studies in Energy, University of Engineering & Technology Peshawar 25000 Pakistan adnan.daud@uetpeshawar.edu.pk; College of Energy, Soochow Institute for Energy and Materials InnovationS (SIEMIS), Soochow University Suzhou 215006 China; Key Laboratory of Advanced Carbon Materials and Wearable Energy Technology of Jiangsu Province, Key Laboratory of Modern Optical Technologies of Ministry of Education Suzhou 215006 China; Department of Computer Science, National University of Technology Islamabad 46000 Pakistan; College of Computer, Qassim University Al-Mulida 51431 Saudi Arabia; Department of Electrical Engineering, Military College of Signals, National University of Sciences and Technology (NUST) Islamabad 46000 Pakistan

## Abstract

Thin film solar cells (TFSCs) suffer from poor light absorption due to their small thickness, which limits most of their practical applications. Surface plasmons generated by plasmonic nanoparticles offer an opportunity for a low-cost and scalable method to optically engineer TFSCs. Here, a systematic simulation study is conducted to improve the absorption efficiency of amorphous silicon (a-Si) by incorporating double sided plasmonic bi-metallic (Al–Cu) nanogratings. The upper pair of the gratings together with an antireflection coating are responsible for minimizing the reflection losses and enhancing the absorption of low wavelength visible light spectrum in the active layer. The bottom pairs are accountable for increasing the absorption of long wavelength photons in the active layer. In this way, a-Si, which is a poor absorber in the long wavelength region, is now able to absorb broadband light from 670–1060 nm with an average simulated absorption rate of more than 70%, and improved simulated photocurrent density of 22.30 mA cm^−2^, respectively. Moreover, simulation results show that the proposed structure reveals many other excellent properties such as small incident angle insensitivity, tunability, and remarkable structural parameters tolerance. Such a design concept is quite versatile and can be extended to other TFSCs.

## Abstract 2

Thin film solar cells (TFSCs) suffer from poor light absorption due to their small thickness, which limits most of their practical applications.

## Graphical abstract

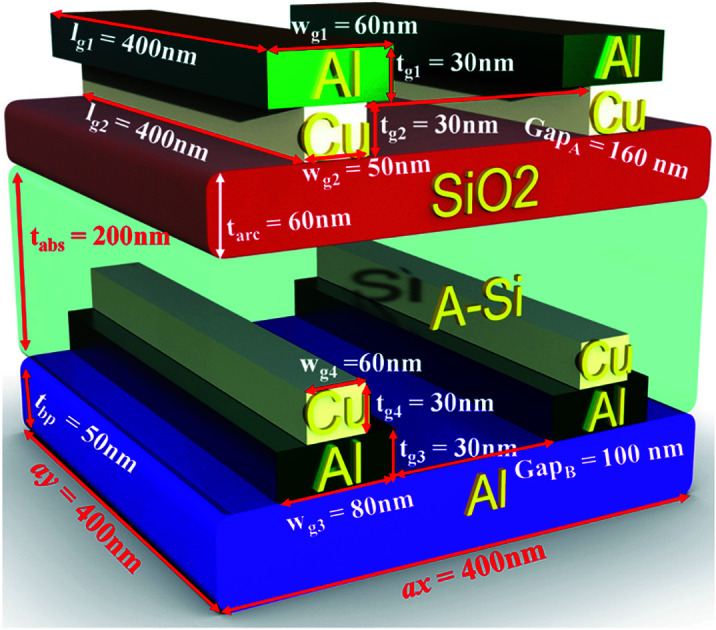

## Introduction

Thin-film solar cells (TFSCs) are some of the most well-known photovoltaic systems, attracting great research attention due to their low cost and flexible substrates.^1–3^ However, when designing TFSCs, one of the main challenges is to effectively improve the absorption of incidence light over a wide range of wavelengths. A key technique for obtaining good light trapping and enhancing the efficiency of solar cells is to engineer the light behavior by using photonic crystals,^4^ diffraction gratings,^5^ anti-reflection coatings,^6^ surface texturing,^7^ and metallic nanoparticles.^3,8^

Plasmonic nanogratings among other structures show a substantial progress as they can be used either as an innovative back reflector patterned on a metal mirror^9–12^ and/or on transparent and conductive oxides (TCO),^13–16^ to improve both the optical path length and optical absorption over a broad spectrum. The effective coupling between metallic nanograting modes and the incident light essentially presents an efficient light trapping developing from surface plasmon resonances and their resultant near-field light concentration.^17–19^ X. Meng *et al.*, proposed a model with different periods of diffraction gratings by combining 1D and 2D front and rear structures within TFSC, based on crystalline silicon and showing spectral density around 90% in the visible range, with a few absorption spikes in the broader spectrum.^20^ However, for better performance, TFSC required uniform absorption enhancement above 90% at longer wavelength. L. Wen *et al.*, analyzed cascaded metal nanogratings and achieved a spectral efficiency above 60% in the wavelength range *i.e.*, 300–750 nm.^21^ However, they could not cover the long wavelength region in the solar spectrum, which indicates high optical losses. Y. Chen *et al.*, presented Ag–Al bi-metallic gratings integrated with a-Si TFSCs by suppressing the loss issues of metal and obtain enhanced absorption from 350 to 750 nm.^22^ However, broadband absorption is not achieved, which makes structure not less appropriate for solar cell applications. Recently, M. Bagmanci *et al.*, proposed an efficient plasmonic structure, which covers ultraviolet and visible range of solar spectrum.^23^ However, the suggested structure has absorption efficiency below 20% at near infrared region, which makes structure not capable to tackle the issue of broadband absorption for TFSCs application. Recently, Z. Khezripour *et al.*, proposed titanium nitride nanogratings on top and bottom of TFSC and obtain broadband absorption at longer wavelengths from 600–1100 nm.^24^ However, they do not cover the rich energy visible portion of the solar spectrum.

Here, we proposed a double-sided hybrid bilayer Al–Cu nanograting structure, which is feasible for TFSC based on a-Si absorber. We examine the corresponding role of both front and back nanogratings and evaluate their influence on the broadband absorption efficiency attainment. The proposed optimized solar cell structure removes the deficiency of a-Si to poorly respond to high wavelength photons and exhibit broadband response with an average absorption of more than 70% over broad spectral range, and enhanced photocurrent density (*J*_sc_) of 22.30 mA cm^−2^.

## Simulation model

**Fig. 1:**
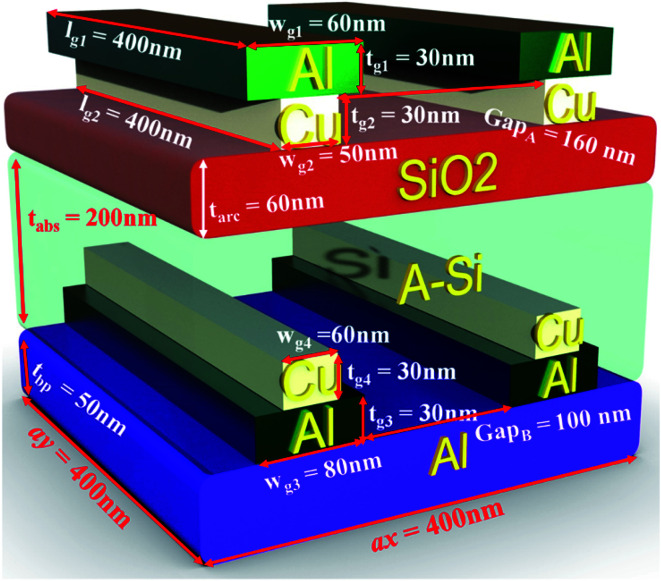
Schematic view of the proposed structure of double side bi-layer gratings.

The proposed a-Si solar cell structure accompanied by front and back side bilayer grating is shown in Fig. 1. The grating is composed of aluminum (Al) and copper (Cu) metals. Since, the plasma frequency of Al is higher than Cu due to high electron density, therefore, it is distinctive that the metal inter-band transition loss in the short-wavelength locality can be muted by placing Al on top with rear Cu grating layer.^25,26^ In our design, the front Al–Cu bilayer metal gratings are used to improve the optical absorption in the active layer due to surface plasmon resonance (LSPR) effect. In contrast to the front surface, at the back side, the inverted version of the metal grating is used along with mirror metal to reflect the transmitted light usually the red to near infrared ranges of the spectrum (700–1100 nm), to the active layer.^27^ The anti-reflective SiO_2_ (ARC) layer is also utilized along with a metal grating for suppressing front side light reflection and serves as a passivation layer due to high band gap.^28^ A very thin absorber layer with thickness of 200 nm, made of a-Si is inserted between plasmonic bilayer nanograting's/ARC and bilayer nanograting's/metal mirror for improved light management. It is notable that numerous research groups have been working on thin film solar cell materials as an absorber layer such as copper indium gallium selenide (CIGS), cadmium telluride (CdTe) with plasmonic nanogratings.^18,29^ However, silicon-based devices show less problems than their contenders such as toxicity and scarcity problems with CdTe cells, and low manufacturing profits of CIGS due to material complexity. Furthermore, a-Si can be produced in a variety of shapes and sizes and greater resistance to heat, as a-Si PV modules experience good results even in high temperatures as observed by National Renewable Energy Laboratory (NREL).^30,31^

The dimensions of the unit cell composed of the proposed structure along *x* and *y* is defined to be *a*_*x*_ = *a*_*y*_ = *a* = 400 nm. The top and bottom Al–Cu resonant bilayer gratings having Al thickness of *t*_g1_ = 30 nm, width *w*_g1_ = 60 nm and length *l*_g1_ = 400 nm, while Cu grating thickness *t*_g2_ = 30 nm, width *w*_g2_ = 50 nm and length *l*_g2_ = 400 nm, respectively. The ARC layer thickness is taken as *t*_arc_ = 60 nm, while that of the active layer a-Si as *t*_abs_ = 200 nm. The thickness of the back-metal reflector is set as *t*_bp_ = 50 nm. The complex dielectric function of Cu, Al, and a-Si are chosen from,^32^ while the dielectric constant of SiO_2_ is taken as 3.9.^33^ The perfectly matched layer thickness of 400 nm is utilized along *z*-direction to eradicate the back reflections. A plane wave illuminates normally on the solar cell with the polarization along *x* direction, which denotes the incident wave propagating along the positive *z*-axis. Floquet periodic boundary conditions are used to repeat the structure along *x* and *y* directions. The environment is chosen to be air of all the simulations and all of them are performed in COMSOL Multiphysics software v5.3 with radio frequency module.

## Results and discussion

**Fig. 2:**
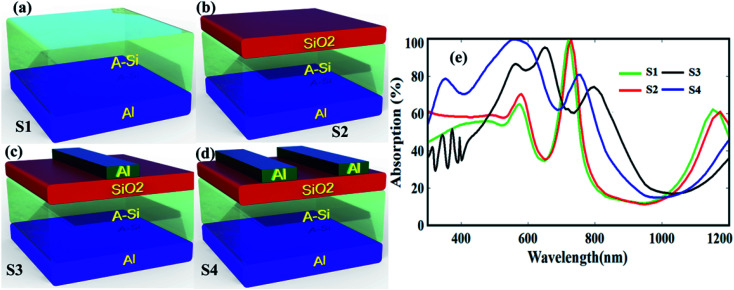
Structure of (a) reference solar cell (S1), (b) ARC layer on top of solar cell (S2), (c) single nanograting and ARC layer (S3), and (d) double nanogratings and ARC layer (S4). (e) Absorption efficiency of S1, S2, S3, and S4 structures, respectively.

To better uncover the physical mechanism of the broadband absorption, we divide the structure into several segments as shown in Fig. 2. The segment S1, contains only Al metal mirror layer, and a-Si absorber layer, see Fig. 2(a); segment S2 consist of SiO_2_ ARC layer on top of absorber layer, see Fig. 2(b); S3 accompanies single Al metal nanograting resonator on top of ARC, see Fig. 2(c); S4 encompasses complete front structure of proposed model having double Al nanograting resonator at the top of a-Si and ARC layer, and a back metal mirror see Fig. 2(d).

Fig. 2(e) shows the measured absorption characteristics of different models. It is found that, the absorption resonance of model S1 is below 60% in the low-wavelength region, while exhibit near unity narrow band response from 700 to 760 nm. Moreover, S1 supports very poor performance in the high-wavelength region because a-Si does not absorb light in this region. So, as a whole, the average absorption efficiency of S1 over broader spectrum is very low due to strong front and back surface reflections, which will not generate enough electron–hole pairs. However, S2 shows slightly better response because of ARC layer. By adding single metallic nanograting (S3), the absorption efficiency comparatively enhances from 520 to 980 nm as indicated by black curve. This is because, metal nanograting harvest the incoming photons by forward scattering or LSPR and the back Al metal foil reflects the transmitted light to the active layer for further absorption.^34–36^ Now, by using double nanogratings (S4), the light absorption is improved approximately 13% in the wavelength region from 300 to 800 nm because the surface plasmons of both the gratings couple strongly and enhanced the absorption in the a-Si layer.^37–39^

**Fig. 3:**
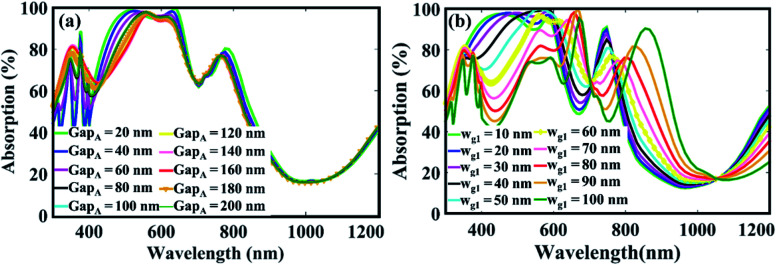
Influence of (a) gap, and (b) width of nanogratings on absorption efficiency.

Since, the LSPR strongly depends upon the geometric parameters and the gap between the nanogratings, therefore, to tune the absorption efficiency in the solar spectrum, we varied the gap and width *w*_g1_ of both the gratings as shown in Fig. 3. It appears that the variation in the gap region slightly affect the absorption in the low wavelength region as shown in Fig. 3(a). This is because, the incoming field uniformly couples to the nanogratings for every gap value. The impact of *w*_g1_ on the absorption profile is very large as shown in Fig. 3(b). For small values of *w*_g1_, the absorption characteristics in the low wavelength is large, and in the high wavelength region, it is small, whereas for large values, the reverse has occurred. This is because, by changing the size, the coupling of the surface plasmons supported by the gratings interfere constructively and destructively in various region in the spectrum.

**Fig. 4:**
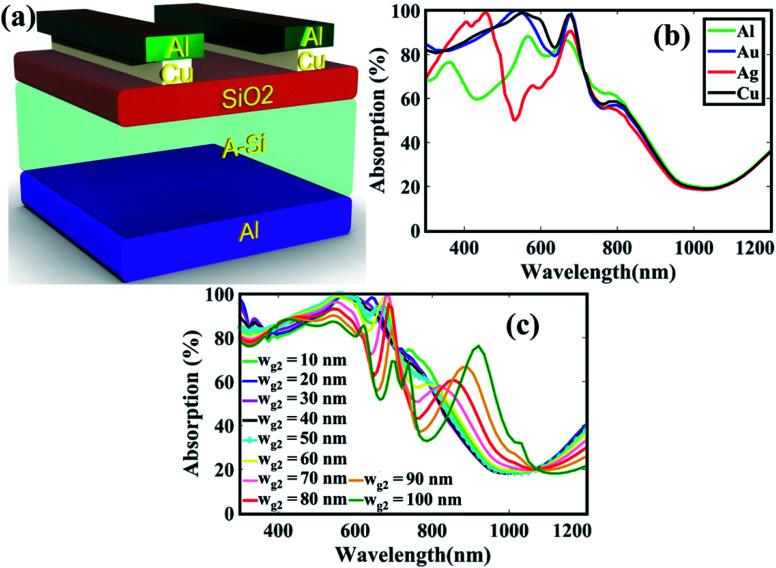
(a) Schematic view of bilayer grating structure (S5), (b) absorption spectra for nanograting materials, and (c) different with of bottom gratings.

In order to further improve the absorption spectral efficiency over broader spectrum, we placed a copper (Cu) grating beneath the Al as shown in Fig. 4(a). So, the Al–Cu bilayer gratings with Cu gratings in contact with the SiO_2_ layer and Al gratings on top of Cu may be able to harvest the solar photons in both the long and short wavelength regions. Moreover, the fabrication of bilayer gratings is not complex because metal alloys or composites have presented higher degree freedom in the design for plasmonic harvesting. For instance, solar cell with multimode Au/Ag bimetallic nanostructures showed an improvement of power conversion efficiency from 6.72% to 7.70%.^40^ In order to check whether Cu is best to be paired with Al gratings, we tested different materials for the bottom gratings with fixed *w*_g2_ = 50 nm as shown in Fig. 4(b). The absorption characteristics indicate that Cu supports superior absorption efficiency and therefore it is preferred to be paired with Al nanogratings. In Fig. 4(c), we varied the widths of the Cu gratings to further optimize the structure. It appears that the absorption efficiency slightly improves compared to single layer gratings because due to high plasma frequency, the top Al gratings suppress the parasitic interband transition loss. In addition, among all the values, *w*_g2_ = 50 nm is the optimum value for the Cu gratings because it exhibits better response in the wavelength range 400–700 nm.

**Fig. 5:**
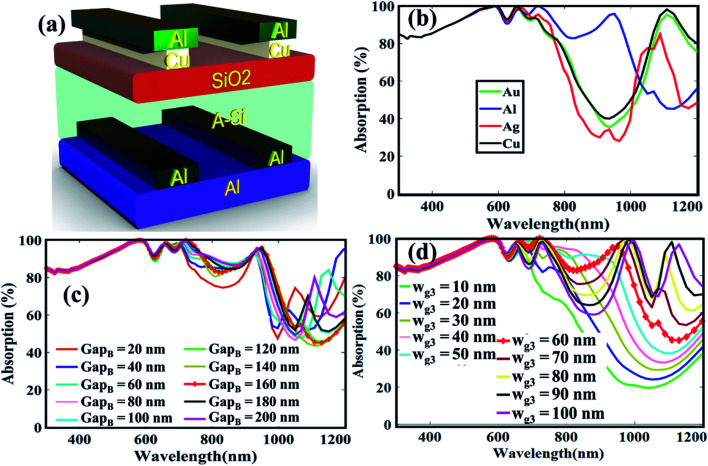
(a) Schematic view of bottom single layer grating structure S6. Absorption spectra for (b) different plasmonic nanograting materials, (c) different gaps between the bottom gratings, and (d) width of nanogratings.

Since, the absorption profile at the long wavelength region is still very poor, therefore, we select the optimized structure S5 and introduced double metal gratings at the top surface of bottom Al metal foil as shown in Fig. 5(a). This structure is named as S6 and the additional gratings will improve the absorption of long wavelength photons which usually reflects from the back Al foil. At first, we tested different materials for the bottom gratings and compare their absorption features as shown in Fig. 5(b). It is found that Al gratings essentially exhibit much better response both in the short and long wavelength regions. Therefore, Al gratings are preferred to be used at the bottom side. Next, the influence of the nanogap between the bottom gratings is studied and again, the absorption efficiency approximately remains the same as shown in Fig. 5(c). So, this indicates that the proposed design is almost independent of the gap spacing between the gratings. The width *w*_g3_, which strongly affect the absorption resonances, are also varied as shown in Fig. 5(d). Here, between 980–1200 nm, the width values from 80–100 nm are suitable due to large absorption enhancement, however, such values of the width exhibit poor absorption efficiency from 770–980 nm, which is a rich energy portion in the solar spectrum. Therefore, in order to maintain uniformity, we select *w*_g3_ = 60 nm, which is the same as the front Al gratings and such value of the width comparatively shows better response.

**Fig. 6:**
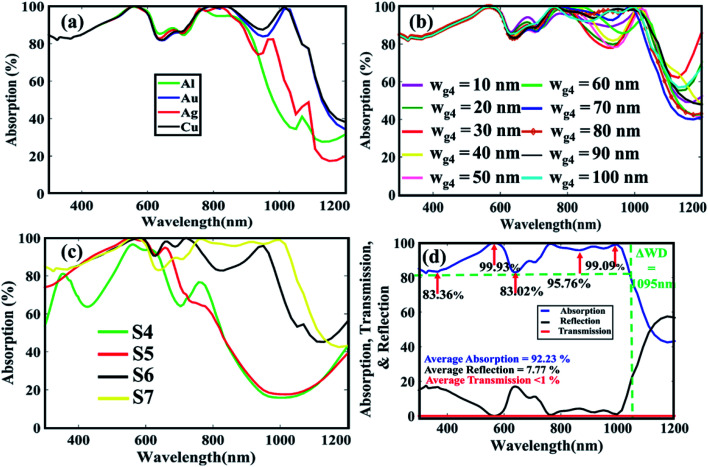
Absorption efficiency for different (a) nanograting materials, and (b) nanograting widths. (c) Comparison of absorption efficiencies of S4, S5, S6, and S7 structures. (d) Absorption, transmission and reflection spectra of double-sided bilayer gratings structure (S7).

Finally, a Cu grating has been placed at the top of Al grating at the rear side as shown in Fig. 1. We named this structure as S7. This bilayer nanogratings at the back side will reflect the transmitted light usually the red to near infrared ranges of the spectrum (700–1100 nm), to the active layer, which will further improve the light absorption. Again, we tested different materials for the newly placed grating, and it is found that Cu supports better absorption features as shown in Fig. 6(a). The impact of different values of the width *w*_g4_ on the absorption efficiency is also studied, which is approximately the same as the previous structures as shown in Fig. 6(b). Therefore, to maintain structure symmetry, the material and dimensions of the new gratings are selected the same as the top gratings. Furthermore, the average absorption efficiency of the structure S7 is more than 92.23% over a broad solar spectrum from 300–1060 nm, which is quite high than the previous structures S4, S5, and S6 as highlighted in Fig. 6(c). Around 25% improvement is observed in S7 compared to S4 solar cell. We also calculated the transmission, reflection and absorption spectra of the double-sided bilayer gratings structure S7 to clearly show the optical losses in the cell. As can be seen in Fig. 6(d), the transmission through the cell is almost zero and the top reflections from the cell is only 7.77%, indicating best performance of the solar cell.

**Fig. 7:**
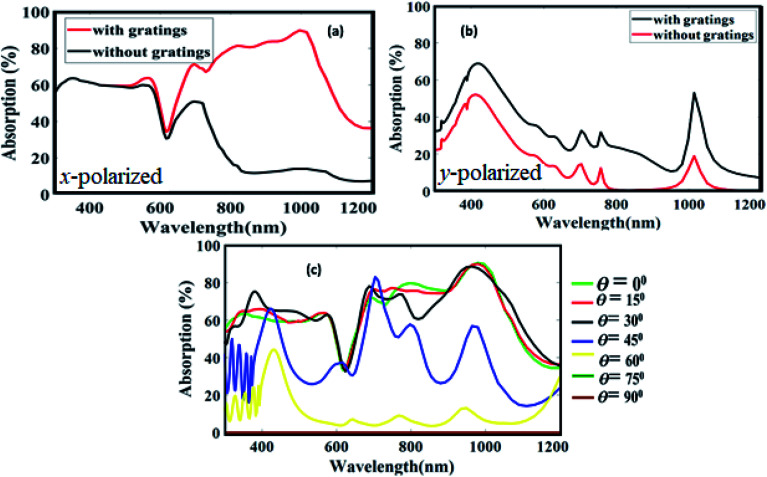
Absorption spectra in a-Si layer with and without nanogratings for (a) *x*-polarized light, and (b) *y*-polarized light, and (c) different incident light angles.

The effect of polarization plays an important role in the performance of solar cells because in general, light for solar cells is polarized in random directions.^41^ Therefore, in order to see the significance of incident light polarization and also to analyze the actual role of the grating elements, we took the structure S7 and calculated the absorption characteristics in the active a-Si layer. Fig. 7(a) shows the absorption spectra of the *x*-polarized light, which is calculated with and without nanogratings. It appears that the cell without gratings exhibit poor response in the long wavelength region, which is improved in greater amount by introducing grating. Again, this is due to the fact that the bilayer grating arrangement can overwhelm the on-resonance parasitic absorption loss in metals at the longer wavelengths because of the small imaginary part of Cu dielectric function and suppress the interband transition loss in the smaller wavelength region due to the large plasma frequency of Al. As a result, approximately 40% improvement in absorption is attained in case of plasmonic nanograting-integrated a-Si solar cell compared to without nanogratings in the long wavelength region. In Fig. 7(b), we have considered the *y*-polarization case and calculated the absorption profile in the active layer for with and without nanogratings. Here, due to geometry of nanogratings, the incident light couples inefficiently to the solar cell compared to the *x*-polarized case, which results in week absorption in the spectrum. Apart from this, it is well known that the angle between the incoming photons and the normal on the solar cell surface plays an important role in the solar cell performance. At different time of the day, before noon to evening, sun rays fall on the solar cell with various angles and potentially affects the trapping of photon inside solar cell. Therefore, in order to illustrate the performance of the proposed solar cell, we varied the incident angles *θ* and measured the light absorption efficiency in the active layer for the *x*-polarized case as shown in Fig. 7(c). It is observed that even for higher value of *θ i.e.*, *θ* = 45^ο^, the structure exhibit broadband absorption resonance, which indicates that the proposed structure is highly suitable for practical applications. Moreover, to quantify the performance of the proposed cell over the entire solar spectrum, we calculated the photocurrent density under the assumption of unity internal quantum efficiency.^42,43^ The expression is1
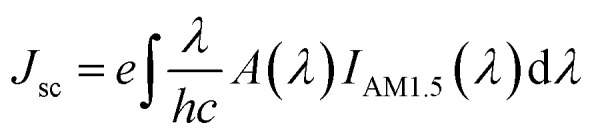
where *e* is the electron charge, which is equal to 1 eV (1.602 × 10^−19^ C), *λ* is the incident light wavelength, which ranges from 300–1200 nm, *h* is the Planck's (*h*) constant, which is *h* = 6.626176 × 10^−34^ joule-seconds, and *I*_AM1.5_(*λ*) is the solar irradiance for Air Mass (AM)1.5. The calculated *J*_sc_ value for the proposed model is 22.30 mA cm^−2^, which is high than the structure without gratings (16.46 mA cm^−2^).

## Conclusion

In summary, novel light harvesting double sided hybrid bi-metallic Al–Cu nanograting structure for thin film a-Si solar cell is investigated. We demonstrated that each grating structure contribute to surface plasmons, which couples and enhance the light absorption in the active layer in greater amount. We have shown that the top metal gratings are responsible to reduce the reflection losses from the top surface and enhance the transmission of photons in the absorber medium, while the role of the bottom nanogratings are to trap the incident light at long wavelengths into the active layer. Therefore, after optimizing the dimensions of the grating structures (*w*_g1_ = *w*_g4_ = 60 nm, and *w*_g2_ = *w*_g3_ = 30 nm), the double sided bi-metallic solar cell structure lead to average broadband absorption of 70% from 670–1060 nm in the a-Si layer and improved *J*_sc_ of 22.30 mA cm^−2^ compared to single sided or double sided single metal gratings. Furthermore, the structure performed well when the incident light angle is varied, which indicates that the proposed solar cell can potentially be employed for practical applications.

## Conflicts of interest

There are no conflicts to declare.

## Supplementary Material
