# Correction: Efficient broadband light absorption in thin-film a-Si solar cell based on double sided hybrid bi-metallic nanogratings

**DOI:** 10.1039/d0ra90042g

**Published:** 2020-04-30

**Authors:** Fazal E. Subhan, Aimal Daud Khan, Fazal E. Hilal, Adnan Daud Khan, Sultan Daud Khan, Rehan Ullah Khan, Muhammad Imran, Muhammad Noman

**Affiliations:** Center for Advanced Studies in Energy, University of Engineering & Technology Peshawar 25000 Pakistan adnan.daud@uetpeshawar.edu.pk; College of Energy, Soochow Institute for Energy and Materials InnovationS (SIEMIS), Soochow University Suzhou 215006 China; Key Laboratory of Advanced Carbon Materials and Wearable Energy Technology of Jiangsu Province, Key Laboratory of Modern Optical Technologies of Ministry of Education Suzhou 215006 China; Department of Computer Science, National University of Technology Islamabad 46000 Pakistan; Department of Information Technology, College of Computer, Qassim University Al-Mulida 51431 Saudi Arabia; Department of Electrical Engineering, Military College of Signals, National University of Sciences and Technology (NUST) Islamabad 46000 Pakistan

## Abstract

Correction for ‘Efficient broadband light absorption in thin-film a-Si solar cell based on double sided hybrid bi-metallic nanogratings’ by Fazal E. Subhan *et al.*, *RSC Adv.*, 2020, **10**, 11836–11842, DOI: 10.1039/C9RA10232A.

The authors regret that the name of one of the authors (Rehan Ullah Khan) was shown incorrectly in the original article. In addition, the affiliations for this author were incorrectly given. The corrected author list and affiliations are as shown above.

The Royal Society of Chemistry apologises for these errors and any consequent inconvenience to authors and readers.

## Supplementary Material

